# Supplementary figures and images for: Genome-Wide Characterization of the Aux/IAA Gene Family in Orchardgrass and a Functional Analysis of DgIAA21 in Responding to Drought Stress
Source: Int J Mol Sci. 2023 Nov 10;24(22):16184. doi: 10.3390/ijms242216184 (PMC10671735; doi:10.3390/ijms242216184)

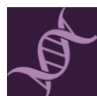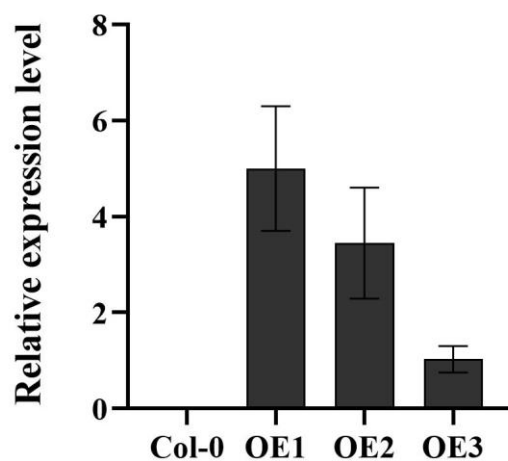

**Figure S1.** Relative expression level of *DgIAA21* gene in Col-0, OE1, OE2 and OE3 Arabidopsis plants.

Supplement: Supplementary file 1 [file ijms-24-16184-s001.zip › ijms-2638822.pdf]
